# Improved Dissolution of Poorly Water-Soluble Rutin via Solid Dispersion Prepared Using a Fluid-Bed Coating System

**DOI:** 10.3390/pharmaceutics17121559

**Published:** 2025-12-03

**Authors:** Hien V. Nguyen, Nga Thi-Thuy Nguyen, Huong Kim-Thien Tran, Thuy Thi-Nhu Huynh, Vi Huyen-Bao Vo, Cuc Thi-Thu Le, Tushar Saha

**Affiliations:** 1 Faculty of Pharmacy, Van Lang University, Ho Chi Minh City 70000, Vietnam; ngwththnga@gmail.com (N.T.-T.N.); tkthuong2002@gmail.com (H.K.-T.T.); 2 Faculty of Pharmacy, Nguyen Tat Thanh University, Ho Chi Minh City 70000, Vietnam; htnthuy@ntt.edu.vn (T.T.-N.H.); vohuyenbaovi@gmail.com (V.H.-B.V.); lttcuc@ntt.edu.vn (C.T.-T.L.); 3 Mastaplex Ltd., Centre for Innovation, University of Otago, Dunedin 9016, New Zealand; tushar.saha21@yahoo.com

**Keywords:** rutin, solid dispersion, fluid-bed coating, dissolution enhancement, stability

## Abstract

**Background/Objectives**: Rutin, a bioactive flavonol glycoside known for its antioxidant, anti-inflammatory, and anticancer activities, faces limited clinical application due to its poor aqueous solubility and low oral bioavailability. This study aimed to enhance the dissolution of rutin by preparing solid dispersions (SDs) using a fluid-bed coating system and formulating the resulting SDs into tablet dosage forms. **Methods**: Rutin was dissolved in methanol and sprayed onto various carriers, including lactose monohydrate, mannitol, microcrystalline cellulose, silicon dioxide, and calcium carbonate. **Results**: Among the carriers tested, lactose monohydrate produced the highest dissolution enhancement, achieving complete drug release within 15 min versus approximately 60% for free rutin. Further investigation into the effect of the rutin-to-lactose ratio on dissolution enhancement identified 1:10 as the most effective. Characterization by powder X-ray diffraction (PXRD) and differential scanning calorimetry (DSC) confirmed a marked reduction in rutin crystallinity, while scanning electron microscopy (SEM) revealed reduced particle size and successful adsorption onto the carrier. Fourier transformed infrared (FT-IR) analysis suggested hydrogen bonding interactions between rutin and lactose monohydrate, which contributed to improved dissolution. The optimal SD was incorporated into tablets containing 50 mg of rutin via wet granulation, and the inclusion of sodium lauryl sulfate further enhanced dissolution. Stability testing demonstrated that the optimized tablets maintained their dissolution profile after 6 months under accelerated conditions (40 °C and 75% RH). **Conclusions**: These findings indicate that fluid-bed coating is an effective approach for preparing SDs to improve the dissolution of rutin and may be extended to other natural polyphenolic compounds.

## 1. Introduction

Poor aqueous solubility presents a significant hurdle for numerous drug candidates, profoundly impacting their absorption and subsequent bioavailability [1]. Since oral administration remains the most convenient and commonly used route for drug delivery, the dissolution of a drug in gastrointestinal fluids is a crucial step for achieving effective absorption [2]. Drugs with low solubility often exhibit incomplete or erratic absorption, leading to suboptimal therapeutic concentrations in systemic circulation and high inter-patient variability [3]. This pervasive problem is estimated to affect nearly 40% of currently marketed drugs and up to 70–90% of compounds under development, underscoring its critical impact on modern pharmaceutical research and development [4].

**Figure 1:**
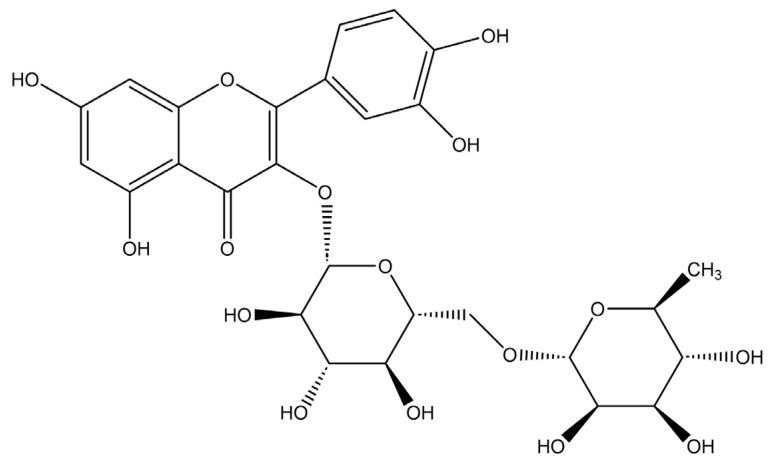
Structure of rutin.

Rutin, a flavonol glycoside identified as 3,3′,4′,5,7-pentahydroxy flavones-3-rutinoside, is also known as quercetin-3-rutinoside or vitamin P. This compound is widely distributed in nature, appearing in plants such as buckwheat, asparagus, and citrus fruits [5]. Structurally, it consists of the flavonol quercetin combined with the disaccharide rutinose, whose molecular structure is shown in Figure 1. This natural compound is renowned for its diverse pharmacological activities, exhibiting antioxidant, anti-inflammatory, and anticarcinogenic properties, making it a promising candidate for various therapeutic applications such as antihypertensive, cardioprotective, neuroprotective, and anti-cancer treatments [6]. Despite its significant therapeutic potential, rutin’s clinical application and oral delivery efficiency are severely limited by its poor aqueous solubility, which poses a substantial hurdle in drug development [7]. Rutin is classified as a Biopharmaceutical Classification System (BCS) class II compound, indicating its low solubility and high permeability [8,9]. For this reason, strategies to enhance rutin’s aqueous solubility are crucial for unlocking its full therapeutic potential and enabling its effective incorporation into pharmaceutical products.

To improve the solubility and dissolution of rutin, various formulation strategies have been explored, including cocrystallization [10], cyclodextrin complexation [11,12], self-emulsifying systems [13], nanoemulsions [14,15], solid dispersions (SDs) [8,16], and nanoparticulate delivery systems [17,18]. Some of which have shown that enhanced solubility and/or dissolution translated into improved oral bioavailability in preclinical animal studies [10,13]. Among these, SD techniques have emerged as an effective approach, involving the dispersion of a drug within a polymeric carrier matrix. This approach is recognized as a promising strategy to enhance the dissolution rate and bioavailability of poorly water-soluble drugs. The improvement in drug dissolution is typically attributed to several mechanisms, such as particle size reduction, enhanced wettability, and the partial or complete conversion of the crystalline drug into an amorphous form [19].

SDs can be prepared using various conventional methods, such as melting or solvent evaporation [20]. In the melting method, the drug and carrier are melted together and then rapidly cooled to solidify the mixture. The solvent evaporation method involves dissolving both components in a common solvent, which is subsequently removed. However, the melting method requires high processing temperatures, making it unsuitable for thermolabile drugs like rutin [21]. Solvent evaporation methods, while avoiding thermal degradation, typically demand large volumes of solvent and often yield SDs with poor flowability and compressibility, leading to operational challenges during large-scale production. Therefore, there is an ongoing need to develop innovative methods to prepare SDs of poorly water-soluble drugs to enhance their scalability and manufacturability. One effective and practical approach is to utilise fluid-bed coating technology for preparing SDs. In this method, the drug is first dissolved in a suitable solvent and then sprayed onto excipients or beads, referred to as substrates [4]. Solvent evaporation and SD deposition occur simultaneously, resulting in the formation of SD granules with improved flowability and compressibility. Another advantage of this approach is that the resulting granules can be easily processed into final dosage forms, such as tablets or capsules, suitable for commercialization. To the best of our knowledge, this approach has not yet been applied to prepare rutin-loaded SDs for enhanced dissolution.

The aims of this study were to (1) improve the dissolution of rutin by preparing the SD using a fluid-bed coating system and (2) to further formulate the prepared SD into a tablet dosage form. The type of carrier and the drug-to-carrier ratio were investigated for their effects on the dissolution enhancement in the SD. The drug crystallinity was significantly reduced in the SD, as evidenced by powder X-ray diffraction (PXRD) and differential scanning calorimetry (DSC) data, together with reduced particle size shown in scanning electron microscopy (SEM) images. Changes in these physical properties can be explained by the intermolecular hydrogen bonding interactions between the drug and the carrier shown in Fourier-transformed infrared (FT-IR) spectra. The prepared SD proved to enhance the dissolution compared to the free drug and was subsequently formulated into a tablet dosage form with enhanced dissolution and a good stability profile over 6 months of storage in accelerated conditions. This scalable SD strategy via fluid-bed coating technology shows potential for improved dissolution of poorly water-soluble rutin, which can subsequently improve its oral bioavailability.

## 2. Materials and Methods

### 2.1. Materials

Rutin (Shaanxi Ruiwo Phytochem Co., Ltd., Ankang, China), lactose monohydrate (Tablettose^®^ 100, Meggle, Wasserburg am Inn, Germany), mannitol (Pearlitol^®^ 50 C, Roquette, Lestrem, France), microcrystalline cellulose (Avicel PH-101, FMC International, Cork, Ireland), silicon dioxide (Syloid^®^ 244 FP, Grace GmbH, Worms, Germany), calcium carbonate (Xilong Chemical, Shantou, China), sodium lauryl sulfate (SLS, Sigma Aldrich, St. Louis, MO, USA), and polyvinyl pyrrolidone K30 (PVP K30, Ashland Chemical, Wilmington, DE, USA) were purchased for use. Distilled water was deionised by reverse osmosis with a Milli-Q water Millipore Purification System™ (Millipore Corp., Bedford, MO, USA). All other chemicals and solvents were of analytical-grade standard.

### 2.2. Equilibrium Solubility Studies of Rutin

The equilibrium solubility of rutin in differing media (solvents, pH buffer solutions, and 1% solutions of surfactants in water) was determined by adding an excess amount of rutin (5 g) to 20 mL of each medium in a 50 mL beaker, followed by magnetically stirring at 500 rpm at 25 °C for 24 h using a ten-position magnetic stirrer (MS-M-S10, DLab Scientific, Beijing, China) to achieve the saturated solution. The filtered aliquot was then analyzed for rutin concentration using a high-performance liquid chromatography (HPLC) system with a UV detector set at a wavelength of 254 nm. The solubility experiments were conducted in triplicate.

### 2.3. Preparation of Solid Dispersions

Rutin-loaded SDs were prepared using the fluid-bed coating technique for a batch size of 330 g. Rutin was dissolved in methanol at a concentration of 70 mg/mL. This solution was then sprayed through a top-spray gun (nozzle diameter: 0.8 mm) at a rate of 0.5 mL/min, with an atomizing air pressure of 0.5 bar, onto the carrier in a fluid-bed coating system (SFBD-1K, Shakti, Ahmedabad, India) at a predetermined drug-to-carrier weight ratio. The inlet air temperature was 60 °C, the inlet air flow was 40 m^3^/h, and the product temperature was 40–45 °C. Following the coating process, the SD was dried for an additional 10 min with an inlet temperature of 60 °C to remove residual solvent. For screening the carriers, five carriers were used, including lactose monohydrate, mannitol, silicon dioxide, calcium carbonate, and microcrystalline cellulose, which were used at a drug-to-carrier ratio of 1:10. To investigate the effect of the drug-to-carrier weight ratio on the dissolution profile of the rutin-loaded SDs, the optimal carrier was used to prepare the SD at a drug-to-carrier ratio of either 1:7.5 (*w*/*w*), 1:10 (*w*/*w*), or 1:12.5 (*w*/*w*).

### 2.4. Powder X-Ray Diffraction (PXRD)

The PXRD diffractograms of free rutin, the SD carrier, and the SD powder were obtained using a Bruker D2 Phaser PXRD system (Bruker, Billerica, MA, USA). A Cu Kα radiation was applied at 30 kV and 10 mA. The sample was scanned in 0.02°, and the data were collected at a 2θ range of 10° to 80°. The data were analyzed using the DIFFRAC.EVA diffraction version 4.2 software (Bruker).

### 2.5. Differential Scanning Calorimetry (DSC)

Thermal analyses of free rutin, the SD carrier, and the SD powder were performed on a NETZSCH DSC Polyma 214 system (NETZSCH-Gerätebau GmbH, Munich, Germany). The sample was contained in a Netzsch Concavus pan with a pierced lid, and a nitrogen flow rate of 40 mL/min was maintained to ensure an inert atmosphere. The sample, ranging from 5 to 10 mg, was heated from 27 °C to 270 °C at a constant rate of 10 °C/min, with an empty pan used as a reference.

### 2.6. Fourier Transformed Infrared Spectroscopy (FT-IR)

FT-IR spectra of rutin, the SD carrier, and the SD powder were recorded using a Varian 3100 FTIR Excalibur Series spectrometer (Varian Inc., Palo Alto, CA, USA) using the transmission mode. The wavelength was scanned from 4000 to 400 cm^−1^ with 16 scans at a resolution of 4 cm^−1^.

### 2.7. Scanning Electron Microscopy (SEM)

The surface morphology of rutin, the SD carrier, and the SD powder were examined using a SEM system (JSM-IT 200, JEOL, Tokyo, Japan). The samples were mounted on a metal stub and sputter-coated with gold under vacuum prior to observation to enhance conductivity. Images were acquired at either 30,000× or 10,000× magnifications operating at an acceleration voltage of 10 kV.

### 2.8. Formulation and Preparation of Rutin-Loaded Tablets

The optimal SD formulation was formulated into tablets containing 50 mg of rutin using the wet granulation method. The binder solution was prepared by dissolving PVP K30 and SLS (if included) in water at a PVP K30 concentration of 12% (*w*/*v*). Rutin, either in free form or SD form, was mixed with the diluents (lactose monohydrate, Avicel PH-101), and the mixture was then sieved through a 0.5-mm sieve. The binder solution was kneaded with the mixture to obtain a homogeneous wet mass, which was then sifted through a 1-mm sieve to obtain wet granules. The resulting granules were then dried at 60 °C for 2 h to obtain dried granules, which were then sifted through a 0.8-mm sieve. The granules were then mixed with croscarmellose sodium and magnesium stearate. This mixture was compressed into a tablet using a rotary tablet press (Shakti Lab Press II, Shakti, Ahmedabad, India) with a concave-faced 13 mm punch to produce tablets with a weight of 900 mg and a target hardness of 35–50 N. The hardness of tablets was determined using a Erweka tablet hardness tester (Erweka GmbH, Heusenstamm, Germany). Tablets also underwent disintegration testing in PBS buffer (pH 6.8) at 37 ± 0.5 °C using a Guoming-BJ2 disintegration apparatus (Guoming, Tianjin, China).

### 2.9. In Vitro Dissolution Testing

Dissolution studies were performed in triplicate using a USP dissolution apparatus II (paddle method) with a dissolution tester (Copley Dis 800i, Copley Scientific, Nottingham, UK) to assess the drug release profiles of free rutin, the prepared rutin-loaded SD powders or tablets, containing 50 mg of rutin. The dissolution medium consisted of 900 mL of phosphate buffer (pH 6.8) containing 0.3% (*w*/*v*) SLS maintained at 37 ± 0.5 °C, with the paddle speed set at 100 rpm. At predetermined time intervals, 5 mL aliquots were withdrawn and immediately replenished with an equal volume of fresh dissolution medium to maintain a constant volume. The withdrawn samples were then filtered through a 0.45-µm syringe filter (Millipore, Merck, Darmstadt, Germany) and analysed for rutin content using a HPLC system with a UV detector set at a wavelength of 254 nm.

### 2.10. Stability Studies

The stability of the optimized rutin tablet formulation was assessed under accelerated conditions in an ICH110 stability chamber (Memmert, Schwabach, Germany) for a period of six months. The tablets were stored in a sealed plastic bottle with a silica desiccant pack. Samples were stored at 40 °C and 75% RH and periodically analyzed for their dissolution profiles to detect any significant changes in dissolution after 3 and 6 months of storage.

### 2.11. HPLC Analysis

The concentration of rutin in the prepared solution was quantified using HPLC, employing a Gemini (C18, 250 × 4.6 mm, 5 µm) analytical column (Phenomenex, Torrance, CA, USA) maintained at 25 °C. A mixture of methanol, phosphate buffer, and tetrahydrofuran at a volume ratio of 1:7:2 (*v*/*v*/*v*) was used as the mobile phase with a flow rate of 1 mL/min. The phosphate buffer was prepared by dissolving 15.6 g monobasic sodium phosphate in 1000 mL of distilled water, with its pH subsequently adjusted to 3.0 using diluted phosphoric acid. Detection of the column effluent was performed using a UV detector set at a wavelength of 254 nm. Prior to use, the mobile phase underwent filtration through a 0.45-mm membrane filter and was degassed. Samples were then injected at a volume of 20 μL into the HPLC system (Shimadzu LC-2050, Shimadzu, Kyoto, Japan) for analysis. This HPLC method was validated for linearity and precision according to the International Council for Harmonisation (ICH)-Q2(R1) guidelines. It showed linearity over a calibration range of 2.5–20 µg/mL, with a coefficient of determination (R^2^) of 0.9999. The Relative Standard Deviation (RSD) values for intra- and inter-precision were 0.96% and 1.37%, respectively.

### 2.12. Statistical Analysis

Statistical analyses were conducted using GraphPad Prism version 8.0 (GraphPad Software Inc., San Diego, CA, USA). Comparisons between two treatment groups were performed with the unpaired Student’s *t*-test, and a *p*-value < 0.05 was considered statistically significant.

## 3. Results

### 3.1. Solubility Studies of Rutin

**Figure 2:**
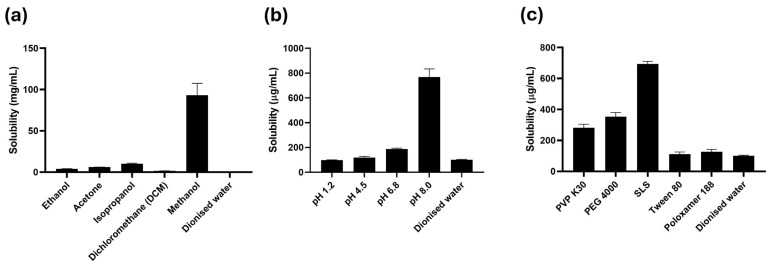
Solubility of rutin in (**a**) organic solvents, (**b**) buffer solutions, and (**c**) 1% (*w*/*v*) surfactant solutions. Data are presented as the mean ± S.D. (*n* = 3).

The saturation solubility of rutin was investigated in various organic solvents, buffer solutions at different pH levels, and 1% (*w*/*v*) surfactant solutions. These studies involved adding an excess amount of rutin to each solvent system, equilibrating the mixtures for 24 h to ensure saturation, followed by filtration and quantification of the dissolved rutin concentration. Figure 2a compares the solubility of rutin in differing solvents, including ethanol, acetone, isopropanol, dichloromethane, methanol, and water. The results indicated that methanol was the most effective solvent for solubilizing rutin among those investigated. The solubility of rutin in methanol was over 900 times greater than in water. Consequently, methanol was chosen to dissolve rutin for subsequent formulation into SDs prior to being subjected to the fluid-bed coating process. Figure 2b illustrates the solubility of rutin in different buffer media at pH 1.2, pH 4.5, pH 6.8, and pH 8.0, as well as in water. The solubility of rutin remained similarly low across the pH range of 1.2 to 6.8, but it increased almost four-fold at pH 8.0. These findings suggest the importance of enhancing rutin’s dissolution at pH levels between 1.2 and 6.8 to improve its bioavailability. Therefore, pH 6.8 was selected as the dissolution medium for testing rutin-loaded formulations. Further investigation into the solubility of rutin in various 1% (*w*/*v*) surfactant solutions in water provided critical insights into potential excipients for enhancing its dissolution. These surfactants could potentially improve the wetting and solubilisation of rutin, offering a viable strategy to overcome its inherent poor aqueous solubility when incorporated into formulations. Figure 2c demonstrates that adding 1% of either PVP K30, PEG 4000, or SLS remarkably enhanced rutin’s solubility, whereas the addition of Tween 80 or Poloxamer 188 did not show a significant improvement compared to deionised water. Among the surfactants tested, SLS was most effective at increasing rutin’s solubility. As a result, SLS was chosen to be added to the dissolution media at a concentration of 0.3% (*w*/*v*) for dissolution studies. Additionally, SLS was selected for inclusion in the rutin-loaded tablet formulations during the downstream tablet formulation design for further dissolution enhancement of rutin.

### 3.2. In Vitro Dissolution Studies of Rutin-Loaded Solid Dispersions

**Figure 3:**
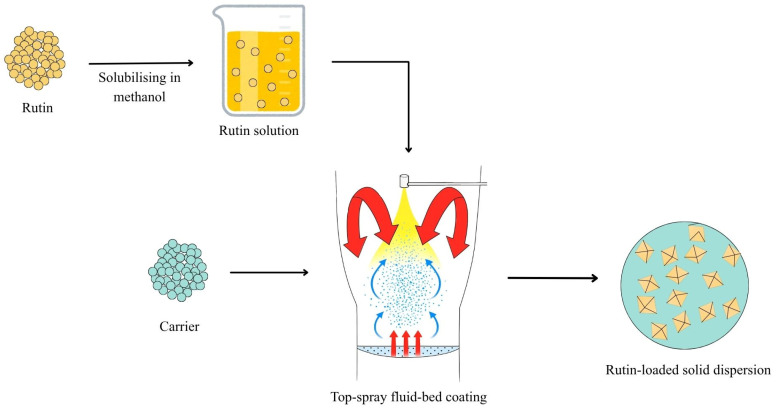
Graphical illustration of the preparation process of rutin-loaded solid dispersions (SDs) using fluid-bed coating technology.

Having determined methanol as the solvent to solubilize rutin, the rutin-loaded SDs were prepared. This process involved dissolving rutin in methanol, followed by top-spray fluid-bed coating the solution onto a carrier, with simultaneous solvent removal within the same fluid-bed system, as graphically illustrated in Figure 3.

A range of SD carriers was compared for their capability of enhancing the dissolution of rutin in the SD system. These carriers included lactose monohydrate, mannitol, microcrystalline cellulose (Avicel PH-101), silicon dioxide, and calcium carbonate. To enable the comparison between different carriers, the SD powders (F1–F5) were prepared using the same rutin-to-carrier weight ratio of 1:10, whose compositions are summarised in Table 1.

**Figure 4:**
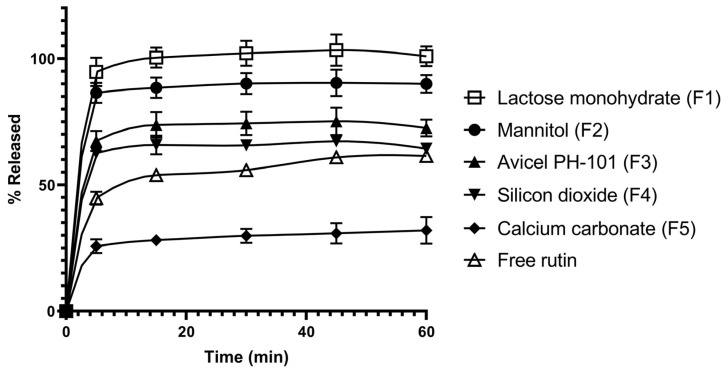
Dissolution profiles of the rutin-loaded solid dispersions prepared from various carriers. Data are presented as the mean ± S.D. (*n* = 3).

The dissolution profiles of these SD powders, as compared to that of pure rutin, are shown in Figure 4. It was found that, except for calcium carbonate-based SD which showed a reduction in the dissolution of rutin compared to free rutin, the other carriers showed an improvement in rutin dissolution with differing levels. The dissolution of rutin at 60 min in SD systems increased in the order: silicon dioxide < Avicel PH-101 < mannitol < lactose monohydrate. Due to the highest level of dissolution enhancement obtained for the SD prepared using lactose monohydrate, which showed complete dissolution after 15 min of dissolution testing compared to ~50% for pure rutin, this carrier was used for further formulation design and characterisation studies.

**Figure 5:**
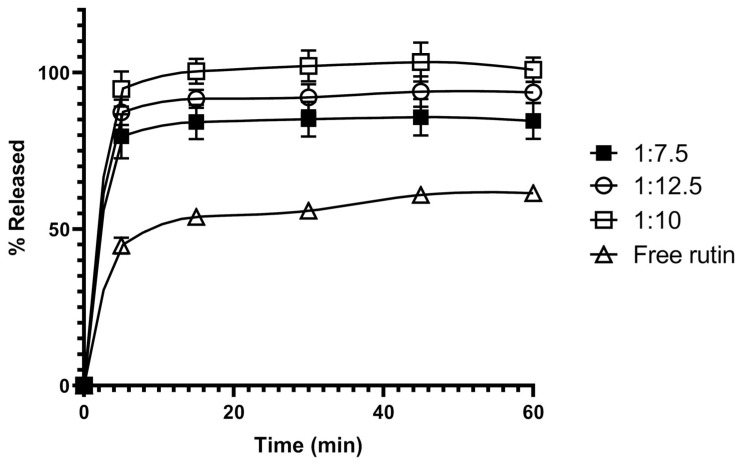
Effect of the rutin-to-lactose weight ratios on the dissolution of the rutin-loaded solid dispersions. Data are presented as the mean ± S.D. (*n* = 3).

The effect of the rutin-to-lactose monohydrate ratio on the dissolution rate of rutin from the SD was then investigated. Three ratios, specifically 1:7.5, 1:10, and 1:12.5 (*w*/*w*), were compared for the dissolution of rutin-loaded SDs, as shown in Figure 5. Increasing the ratio from 1:7.5 (F6) to 1:10 (F1) showed a significant increase in the dissolution of rutin at 60 min (*p* = 0.015), from approximately 84% to 100%. However, a further increase from 1:10 (F1) to 1:12.5 (F7) did not result in an additional increase in drug dissolution. Therefore, the SD prepared using lactose monohydrate as the carrier with a drug-to-carrier ratio of 1:10 was chosen as the optimal SD formulation for characterisations and for preparing the SD-based tablet dosage form.

### 3.3. Powder X-Ray Diffraction (PXRD)

**Figure 6:**
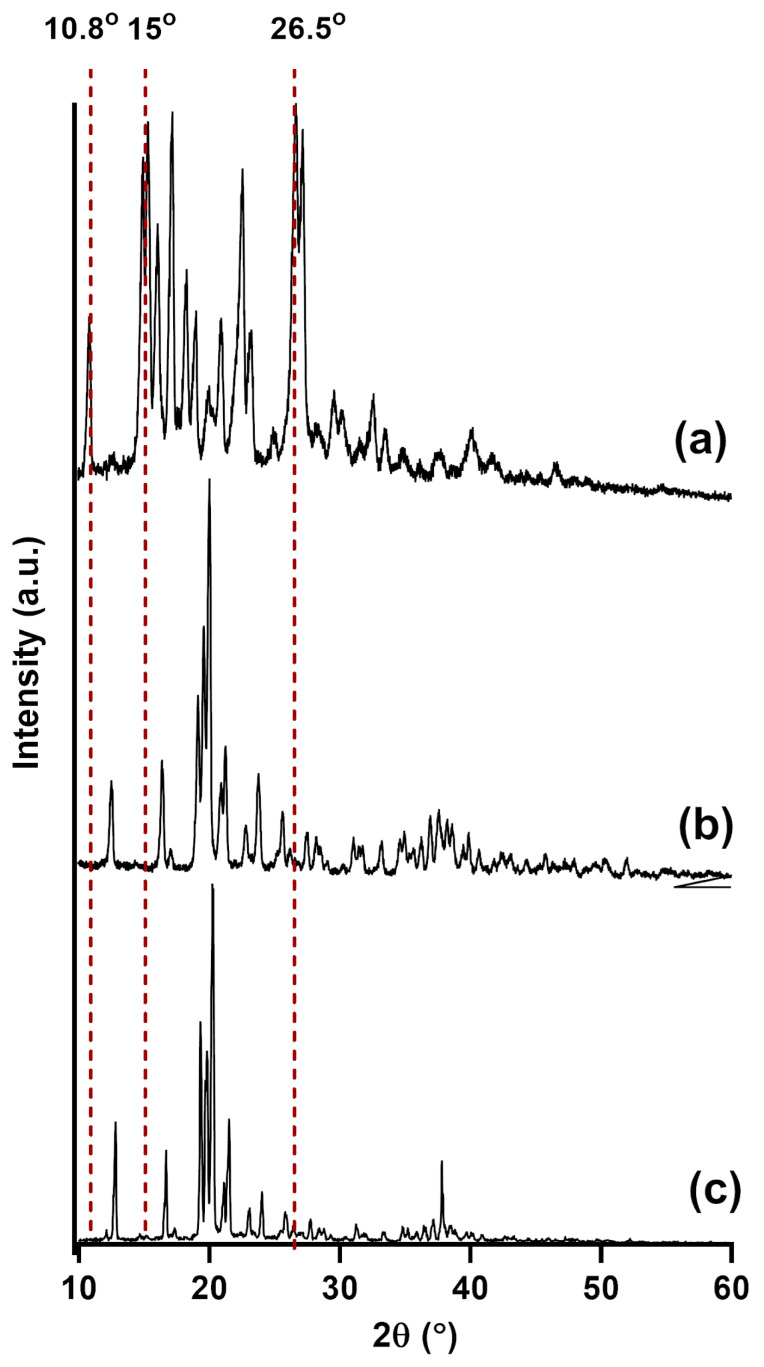
PXRD diffractograms of (**a**) free rutin, (**b**) lactose monohydrate, and (**c**) rutin–lactose solid dispersion (F1).

To further elucidate the morphological state of rutin within the optimized SD, PXRD analysis was performed to assess its crystallinity in comparison to pure rutin and lactose monohydrate, as shown in Figure 6. The PXRD diffractogram of pure rutin has multiple peaks, such as sharp peaks at 2θ angles of 10.8°, 15°, or 26.5°, which are characteristic of its crystalline nature. The PXRD diffractogram of lactose monohydrate showed sharp peaks at 16.0°, 19.8°, and 23.6°. The diffractogram of the optimized rutin-loaded SD exhibited a significant reduction in these characteristic peaks of rutin while maintaining the peaks belonging to lactose monohydrate, suggesting a significant decrease in rutin’s crystallinity within the SD.

### 3.4. Differential Scanning Calorimetry (DSC)

**Figure 7:**
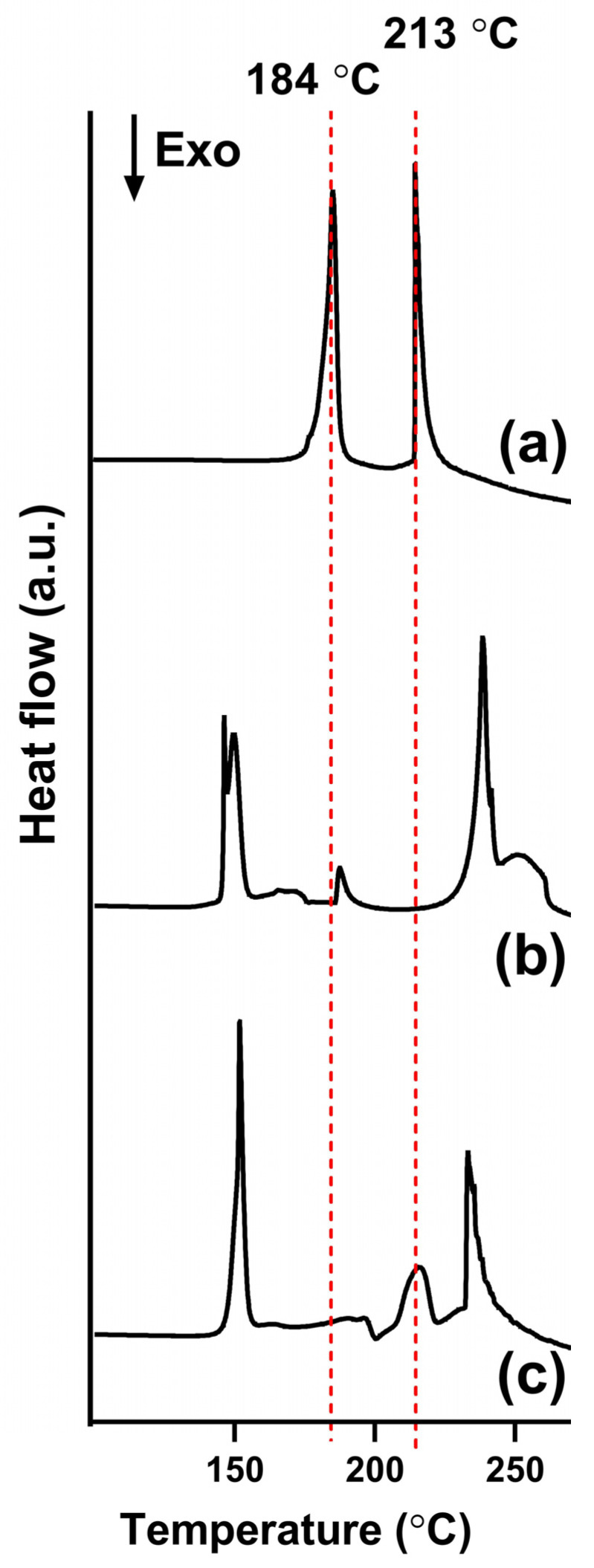
DSC thermograms of (**a**) free rutin, (**b**) lactose monohydrate, and (**c**) rutin–lactose solid dispersion (F1).

DSC analysis further supported these findings obtained by PXRD by analyzing the thermal behavior of rutin, the carriers, and the SD, revealing changes in melting points and enthalpies. Compared to PXRD, DSC is more sensitive for detecting crystalline material with a smaller crystalline size [22]. The thermogram of pure rutin displayed two endothermic peaks at around 184 °C and 213 °C (Figure 7a), while that of lactose monohydrate showed sharp peaks at 145 °C and 235 °C (Figure 7b). The DSC thermogram of the SD showed the disappearance of the peak at 184 °C, while the peak at 213 °C had a significant reduction in peak intensity compared to that of pure rutin (Figure 7c). This observation consistently suggests a reduction in the crystallinity of rutin within the SD system.

### 3.5. Scanning Electron Microscopy (SEM)

**Figure 8:**
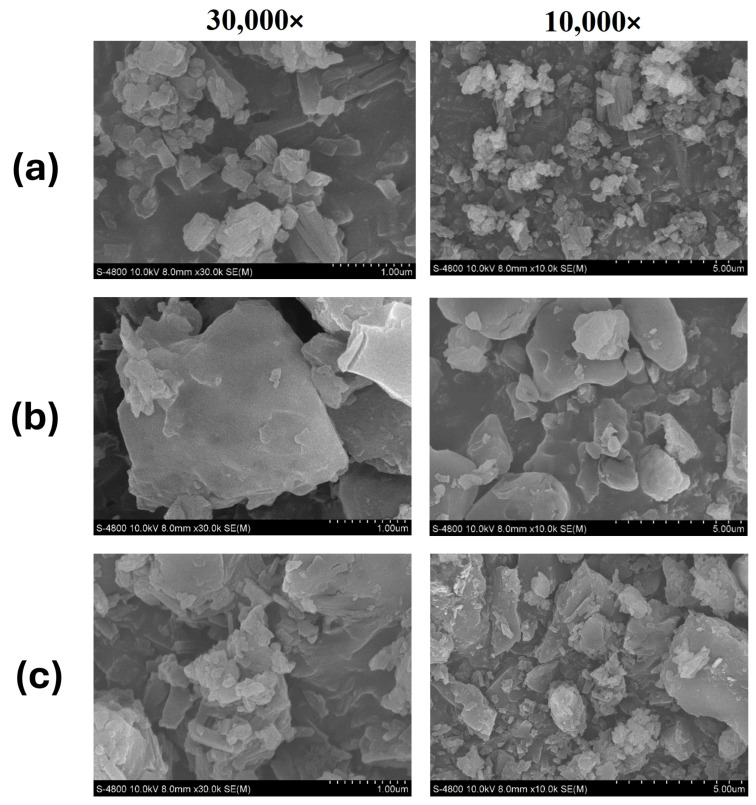
SEM images of (**a**) free rutin, (**b**) lactose monohydrate, and (**c**) rutin–lactose solid dispersion (F1).

SEM was employed to visually examine the surface morphological characteristics and particle size of the optimized SD F1, suggesting insights into its surface topography and the structure of the SD system. SEM images of pure rutin, lactose monohydrate, and the rutin-loaded SD are presented in Figure 8. Pure rutin and lactose monohydrate both appeared as irregularly shaped crystalline particles, with a size ranging from 1 to 10 μm. The surface of lactose monohydrate particles was shown to be relatively smooth. SEM images of the SD, F1 showed that the rutin particles were attached to the surface of lactose monohydrate with a smaller size compared to the original rutin, confirming the adsorption of reprecipitated drugs onto the carrier following the fluid-bed coating process.

### 3.6. Fourier-Transformed Infrared Spectroscopy (FT-IR)

**Figure 9:**
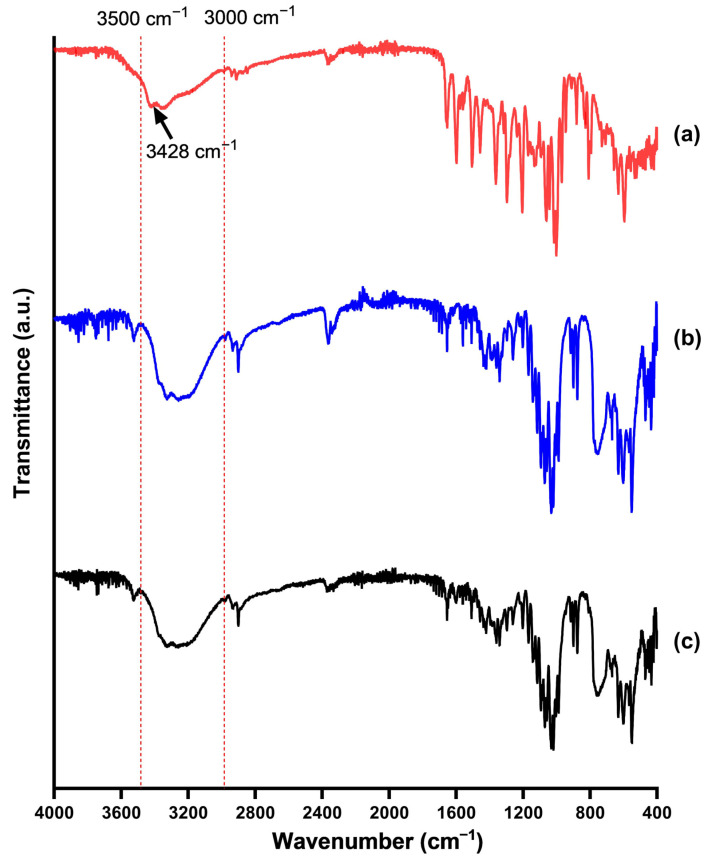
FT-IR spectra of (**a**) free rutin, (**b**) lactose monohydrate, and (**c**) rutin–lactose solid dispersion (F1).

FTIR was utilized to investigate potential intermolecular interactions between rutin and the lactose monohydrate carrier within the SD by analyzing characteristic absorption bands. The FT-IR spectra of pure rutin exhibited characteristic peaks corresponding to its functional groups, such as hydroxyl and carbonyl stretches at 3428 and 1658 cm^−1^, respectively (Figure 9a). Lactose monohydrate displayed a broad characteristic peak between 3000–3500 cm^−1^, attributable to hydroxyl group stretching (Figure 9b). The spectrum of the SD showed the characteristic peaks from both rutin and lactose monohydrate, confirming the presence of both components (Figure 9c). Notably, the peak at 3428 cm^−1^ belonging to the phenolic hydroxyl groups of rutin disappeared in the SD’s FT-IR spectrum. In addition, a noticeable reduction in the intensity of the broad peak ranging from 3000–3500 cm^−1^, corresponding to the hydroxyl groups of lactose monohydrate, was observed in the SD. These changes in the peaks of the phenolic groups of rutin and the hydroxyl groups of lactose monohydrate suggest the formation of hydrogen bonds between these two groups. These molecular-level interactions are believed to contribute to the improved dissolution of rutin.

### 3.7. Formulations and Physicochemical Characterisations of Rutin-Loaded Tablets

Following the development of the rutin-containing SD systems, the subsequent phase involved incorporating this optimized SD into a final tablet dosage form using the wet granulation method, with each tablet containing 50 mg of rutin. Four formulations (T1–T4) were investigated, whose compositions are summarized in Table 2, to understand the impact of the SD system and the inclusion of the solubilizer, SLS, on tablet dissolution. Specifically, T1 and T2 comprised pure rutin, with T2 incorporating SLS in the tablet matrix and T1 omitting it. Conversely, T3 and T4 employed the rutin-loaded SD powder, without and with SLS in the tablet compositions, respectively.

The drug content, hardness, and disintegration time for these tablet formulations are presented in Table 3. All four formulations demonstrated acceptable drug content, ranging from 98% to 103.5%. The hardness of the four formulations was also comparable (40–45 N). The disintegration times of T1, T2, and T3 were similar (~6 min), whereas that of T4 exhibited a delayed disintegration (~8.5 min).

**Figure 10:**
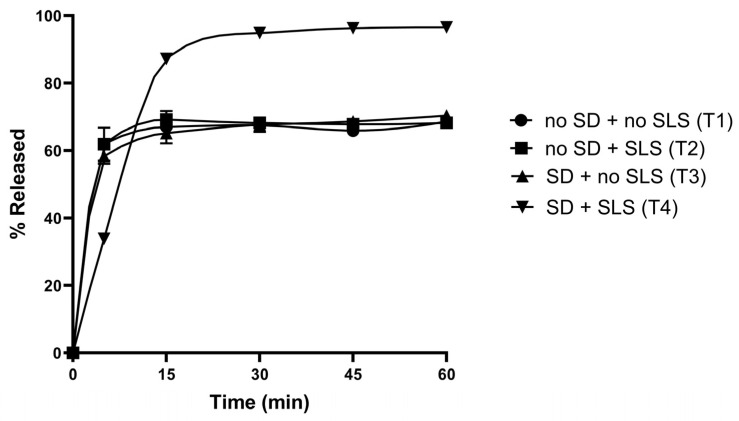
Dissolution profiles of rutin-loaded tablets (T1–T4). Data are presented as the mean ± S.D. (*n* = 3).

The dissolution profiles of these four tablet formulations are depicted in Figure 10. It was observed that tablets containing pure rutin displayed a dissolution rate akin to that of pure rutin powder, with its dissolution at 60 min approximating 60%. This finding suggests that merely incorporating pure rutin into a tablet formulation, even with a solubilizer, does not markedly alter its dissolution characteristics. The tablet formulation T3, which utilized the rutin-loaded SD without the addition of SLS, did not demonstrate any marked dissolution enhancement compared to T1 and T2, and was inferior to that of the SD powder, F1. The formulation T4, which employed the rutin in the form of the SD powder (F1) with added SLS in the tablet matrix, exhibited significantly higher dissolution compared to the other three tablet formulations. Although T4 demonstrated lower dissolution at 5 min, it achieved almost complete dissolution after 15 min, similar to the corresponding F1 powder.

### 3.8. Stability Studies

**Figure 11:**
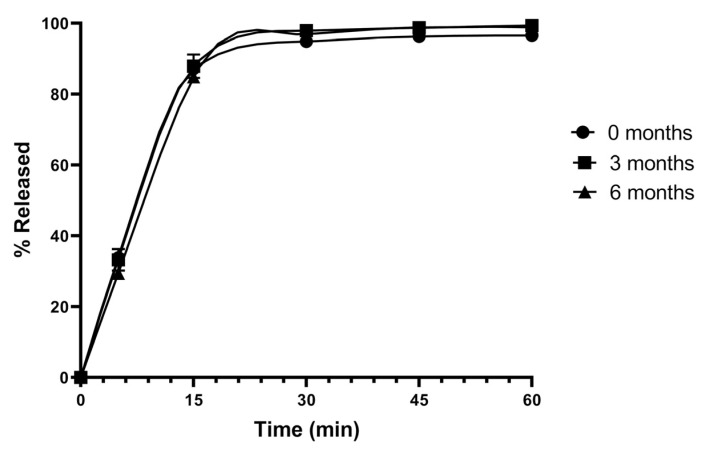
Dissolution profiles of the rutin-loaded tablet, T4 upon storage in the accelerated conditions (40 °C, 75%RH). Data are presented as the mean ± S.D. (*n* = 3).

A disadvantage of SDs is their propensity to recrystallize during storage, which can reduce the dissolution rate [23]. To evaluate the stability of the SD-incorporated tablet formulations, the optimized tablet formulation, T4, was subjected to a stability study, in which the tablets were stored in accelerated conditions (40 °C and 75% RH) for 6 months. After 3 and 6 months, the tablets were evaluated for their dissolution rates, as shown in Figure 11. These results demonstrated no marked changes in the dissolution profiles of tablets following 3 and 6 months of storage compared to the tablets before storage, indicating a good stability profile for the tablet formulation T4.

## 4. Discussion

This research shows that the SD approach prepared via fluid-bed coating technology can effectively enhance the dissolution of the poorly water soluble rutin. The resulting SD was successfully transformed into a tablet dosage form without compromising the drug dissolution rate. In addition to its widespread availability in the large-scale pharmaceutical industry, fluid-bed coating technology addresses limitations of conventional SD preparation methods, such as poor powder physical properties and high production costs. For these reasons, several previous studies have utilised fluid-bed coating technology to prepare SDs [24,25,26]. This technology has also been employed for the preparation of the commercial product Sporanox^®^ to enhance the dissolution of its active ingredient, itraconazole [4]. However, in those previous studies, the SDs prepared by fluid-bed coating techniques are typically a trinary system where the drug and polymer are dissolved in a solvent and is then sprayed onto an inert carrier. In contrast, this research employs the fluid-bed coating technology to prepare the binary systems where drug solution is directly sprayed onto the carrier, which provides a simple and effective system compared to the SD systems prepared previously.

The solubility of rutin in different media was determined to select the conditions for preparation and characterisations for the rutin-loaded SDs. Methanol was found to be most effective in solubilising rutin among the solvent tested (Figure 2a), which was consistent with previous work by Krewson et al. [27]. Therefore, this solvent was used to dissolve rutin in the preparation process of SD. The use of methanol for dissolving rutin can therefore minimise the amount of organic solvents, reducing production cost and processing time. However, it is noted that methanol is a toxic organic solvent when taken orally; thus, future studies must determine the residual solvent levels to meet regulatory requirements. SLS was also found to be the most effective solubilizer in enhancing the solubility of rutin (Figure 2c), thus this solubilizer was used to add to the rutin tablet formulations for enhanced dissolution. Regarding the effect of pH on rutin’s solubility, the solubility of rutin was shown to be pH independent in a pH range from 1.2 to 6.8, while being higher at pH 8.0 (Figure 2b). These solubility results can be explained by the acid–base behavior of rutin. In the pH range of 1.2–6.8, which is below its pKa (~7.2), rutin remains largely in the unionized form and thus exhibits similarly low solubility [28]. In contrast, at pH 8.0 that is above its pKa, deprotonation of the phenolic groups occurs, leading to the formation of ionized species with markedly higher solubility. Accordingly, pH 6.8 was selected as the dissolution medium to simulate intestinal conditions where rutin remains poorly soluble in its unionized form. The rationale of adding 0.3% SLS to the dissolution medium is to ensure sink conditions for poorly soluble rutin, which highlights the dissolution enhancement provided by our SD strategy. However, this relatively high concentration of SLS might not fully represent typical physiological conditions. Therefore, future studies could investigate the dissolution performance in more biorelevant media, which more closely mimic the gastrointestinal environment.

The SDs were prepared using the fluid-bed coating system, where rutin was solubilized in methanol, followed by spraying the solution onto various carriers (Figure 3). The carriers selected to prepare the rutin-loaded SDs are common tablet diluents, which are also known to act as adsorbents for the attachment of poorly soluble drug compounds [29,30,31]. Since these excipients are widely used in tablet formulations, the resulting SDs can be readily developed into a tablet dosage form. These carriers can be generally divided into hydrophilic and hydrophobic carriers. Hydrophilic carriers consist of lactose monohydrate, mannitol, and silicon dioxide, while hydrophobic carriers include microcrystalline cellulose and calcium carbonate. All the SD powders were shown to increase the dissolution of rutin, except for the SD prepared with calcium carbonate. Generally, hydrophilic carriers can enhance the dissolution of rutin better than hydrophobic carriers. This finding could be due to the hydrophilic carriers facilitating the wetting of the powder when it contacts the dissolution media. Among the carriers investigated, lactose monohydrate was best at enhancing drug dissolution (Figure 4); therefore, the effect of the drug-to-carrier ratio on drug dissolution was investigated using the lactose monohydrate-based SD system. The SD powder with a rutin-to-lactose monohydrate weight ratio of 1:10 was shown to be optimal for enhanced dissolution (Figure 5). Previous studies have reported improved rutin dissolution using SD systems with carriers, such as poloxamer [8], PVP K30 [16] or silicon dioxide [32]. However, these systems often require large carrier quantities, which can negatively affect powder properties such as flowability, compressibility or disintegration, complicating downstream formulation. In contrast, lactose monohydrate can enhance both disintegration and powder flowability, making it a practical carrier for tablet and capsule formulations [33]. In addition to the effect of formulation parameters on drug dissolution shown in this study, future studies should investigate the impact of critical processing parameters such as spraying speed or atomizing air pressure on dissolution. These factors influence droplet formation, coating uniformity, and drying kinetics during fluid bed processing, which in turn affect particle morphology and drug-carrier interactions [34]. Optimizing these parameters could further enhance the dissolution performance and scalability of the rutin-loaded SDs by fluid-bed coating technology.

The optimal SD formulation was physicochemically characterized to understand the mechanism behind its enhanced drug dissolution. DSC and XRD data (Figure 6 and Figure 7) consistently showed a significant reduction in the crystallinity of rutin in the form of SD compared to the free drug. The transformation toward an amorphous state can enhance the dissolution of poorly water-soluble drugs [35]. Future studies could further strengthen the design by comparing the SD with its corresponding physical mixture, as this would isolate the effects attributable to the SD process from those simply due to the carrier’s presence. SEM images (Figure 8) confirmed the structure of the SD system, in which the reprecipitated drug was adsorbed onto the surface of the carrier upon the fluid-bed coating process. It is also notable that the particle size of reprecipitated drugs in the SD was shown to be smaller than that of the free drugs. Both the reduction in drug particle size and the reduction in crystallinity collectively lead to the enhanced dissolution of rutin. The observed changes in physical state likely arise from strong hydrogen-bonding interactions between rutin and lactose monohydrate, as supported by FT-IR data (Figure 9). These interactions inhibit crystal growth during drying, stabilizing the amorphous state and maintaining small particle size essential for enhanced dissolution [36]. This also suggests that this method could be applied to other polyphenolic compounds possessing numerous hydrogen acceptors and donors in their molecular structures.

The optimal solid dispersion (SD) powder, F1, was selected for tablet formulation to facilitate administration and improve patient compliance [37]. Tablets prepared from the SD powder exhibited lower dissolution than the F1 powder but comparable dissolution to tablets made from the free drug. However, the dissolution rate markedly increased to nearly 100% when SLS was incorporated into the tablet formulation (Figure 10). This result indicates a synergistic effect between SLS addition and SD use in enhancing rutin dissolution, as the absence of either led to suboptimal performance. Although the tablet formulation T4 containing SLS displayed a longer disintegration time, it achieved superior dissolution. The prolonged disintegration time may result from SLS reducing the water uptake rate by modifying the surface wettability of hydrophilic excipients, possibly forming a viscous gel-like layer or decreasing capillary forces that facilitate water penetration into the tablet [38]. The enhanced dissolution, nonetheless, can be attributed to the improved wettability of the compressed powder upon contact with the dissolution medium [39]. The optimal tablet formulation, T4, also showed good stability in accelerated conditions as no significant changes in dissolution profiles were observed (Figure 11). These stability results indicate the robustness of the formulation, suggesting its potential for a commercially viable shelf life, particularly given the inherent challenges of maintaining the amorphous state in SDs [8].

Overall, this study contributes to the growing body of evidence demonstrating that fluid-bed coating is a versatile and scalable platform for developing SDs of poorly water-soluble compounds. A simplified binary SD system utilizing lactose monohydrate as an optimal hydrophilic carrier significantly enhanced rutin dissolution. This improvement can be attributed to the marked reduction in drug crystallinity and the formation of smaller reprecipitated particles, likely resulting from strong intermolecular hydrogen bonding between rutin and lactose monohydrate. The study also highlights the synergistic effects of the SD system and surfactant incorporation when formulated as tablets. Future studies should include in vivo evaluations to validate these findings and characterize the pharmacokinetic profile of the optimized rutin formulation in relevant biological systems. Such investigations are essential for translating enhanced in vitro dissolution into improved therapeutic outcomes.

## Figures and Tables

**Table 1:** Formulation compositions (g) of rutin-loaded solid dispersions.

Composition	Formulation Code						
	F1	F2	F3	F4	F5	F6	F7
Rutin	30	30	30	30	30	30	30
Lactose monohydrate	300	-	-		-	225	375
Mannitol	-	300	-		-	-	-
Microcrystalline cellulose (Avicel PH101)	-	-	300	-	-	-	-
Silicon dioxide	-	-	-	300	-	-	-
Calcium carbonate	-	-	-	-	300	-	-
Total weight (g)	330	330	330	330	330	255	405

**Table 2:** Formulation compositions (mg) of rutin-loaded tablets.

Composition	Formulation Code			
	T1	T2	T3	T4
Rutin	50	50	-	-
Lactose monohydrate	500	500	-	-
Rutin–lactose solid dispersion (F1)	-	-	550	550
Microcrystalline cellulose (Avicel PH101)	269	269	269	269
PVP K30	27	27	27	27
Crosscamellose sodium	45	45	45	45
SLS	-	30	-	30
Magnesium stearate	9	9	9	9
Tablet weight (mg)	900	900	900	900

PVP K30, polyvinyl pyrrolidone K30; SLS, sodium lauryl sulfate.

**Table 3:** Physicochemical properties of rutin-loaded tablets (T1–T4). Data are expressed as the mean ± S.D. (*n* = 3).

Formulation Code	Drug Content (%)	Hardness (N)	Disintegration Time (min)
T1	103.5 ± 1.2	41.3 ± 1.0	5.4 ± 0.5
T2	102.9 ± 0.9	40.8 ± 0.5	6.8 ± 0.2
T3	103.3 ± 0.4	40.6 ± 0.6	5.7 ± 0.5
T4	98.2 ± 0.7	41.6 ± 1.2	8.6 ± 0.7

## Data Availability

The data presented in this study are available on request from the corresponding author.

## Abbreviations

The following abbreviations are used in this manuscript:

SD	Solid dispersion
PXRD	Powder X-ray diffraction
DSC	Differential scanning calorimetry
SEM	Scanning electron microscopy
FT-IR	Fourier transformed infrared
SLS	Sodium lauryl sulfate
PVP K30	Polyvinyl pyrrolidone K30
ICH	International Council for Harmonisation
RSD	Relative Standard Deviation
